# *In Vitro* Whitening Effect of a Hydroxyapatite-Based Oral Care Gel

**DOI:** 10.1055/s-0040-1714759

**Published:** 2020-08-13

**Authors:** Sandra Sarembe, Joachim Enax, Maria Morawietz, Andreas Kiesow, Frederic Meyer

**Affiliations:** 1 Fraunhofer Institute for Microstructure of Materials and Systems IMWS, Halle, Germany; 2 Research Department, Dr. Kurt Wolff GmbH and Co. KG, Bielefeld, Germany

**Keywords:** teeth whitening, hydroxyapatite, oral care, spectrophotometry

## Abstract

**Objective** Oral care formulations aim to prevent oral diseases such as dental caries and gingivitis. Additionally, desire for white teeth still exists across all age groups. It is known that most whitening toothpastes are highly abrasive and can be harmful to teeth and gingiva. Therefore, a gel formulation with biomimetic hydroxyapatite (HAP; Ca _5_ [PO _4_ ] _3_ [OH]) as active ingredient was developed. This formulation was tested with respect to its tooth whitening properties in an *in vitro* study.

**Materials and Methods** Enamel samples were allocated to either group (a) HAP gel, (b) whitening mouth rinse with phosphates, or (c) negative control (distilled water). Test products were applied by finger (a) or were rinsed (b, c) for 1, 3, and 9 (b and c only) cycles, respectively.

**Results** Color changes (ΔE) were measured spectrophotometrically. Group (a) showed a significant increase in color changes with respect to whitening after one cycle (mean ΔE = 5.4 [±2.66], *p* ≤ 0.006) and three cycles (mean ΔE = 11.2 [±3.11], *p* < 0.0001) compared to groups (b) and (c). For group (b), a significant increase in color change was measured after three (mean ΔE = 2.77 [±1.01], *p* = 0.02) and nine cycles (mean ΔE = 3.27 [±1.61], *p* = 0.006) compared to (c). Group (c) showed only minor and statistically insignificant color changes.

**Conclusion** This *in vitro* study demonstrated a significantly higher ad hoc whitening effect of the HAP gel compared to the mouth rinse and water after short-time application.

## Introduction

Oral care products and formulations for teeth whitening have gained an increased attention worldwide. Many people prefer white teeth since its discoloration may affect their quality of life. 1 Besides, professional applications (e.g., bleaching with peroxides and professional dental cleaning), various whitening products for home use are commercially available. This includes, for example, whitening toothpastes, mouthwashes, and gels. 1 2

Whitening formulations often contain abrasive agents (e.g. hydrated silica, alumina, perlite), chemical agents (e.g. phosphates), or optical agents (e.g. blue covarine). 1 2 However, highly abrasive agents and peroxides may induce unwanted side effects. 3 4 Toothpastes with high radioactive dentin abrasion (RDA) values may be harmful to exposed dentin and gingiva. Additionally, the use of peroxides could lead to tooth sensitivity and unwanted damage of the organic matrix of both enamel and dentin. 1 Therefore, research has focused on alternative nonoxidative, and less-abrasive tooth whitening agents. 5 One of these agents belong to the group of calcium phosphates like particulate hydroxyapatite (HAP; Ca _5_ [PO _4_ ] _3_ [OH]). 1 5 6 7 8 9 In particular, biomimetic HAP is inspired by structure and composition of natural enamel crystallites 10 and shows a broad range of applications in preventive oral health care, 11 12 13 14 15 16 17 such as antibiofilm properties. 18 A recently published in vivo study has shown its positive properties to release calcium in dental biofilms. 19 This can be helpful to buffer cariogenic biofilms, and with increased calcium-levels leading to a shift from tooth demineralization to remineralization. 11 17

The aim of this in vitro study was to test the whitening effect of a newly developed gel formulation based on microcrystalline HAP. To exclude any abrasive influence by toothpaste abrasives harder than natural enamel, 20 and more importantly by the toothbrush that is used in daily oral care, this HAP-gel formulation does not contain commonly used abrasives (e.g. hydrated silica) and was applied by finger. This fact is of importance since the whitening effect of the toothpaste is mainly determined by abrasive ingredients and the mechanical cleaning efficacy of the toothbrush. 1 The assumed mode of action of the newly developed whitening formulation with HAP is based on its adhesion of particles on the enamel surface.

Consequently, the focus of this in vitro study was the analysis of the specific whitening effect of a HAP-containing containing gel. This would be helpful for individuals who prefer a brighter appearance of their teeth but are suffering from dentin hypersensitivity or patients suffering from gingivitis or periodontitis. Our hypothesis was that HAP would change the appearance of the natural tooth color to a brighter (whiter) color without having abrasive and/or oxidizing properties. A nonabrasive, commercially available whitening mouth rinse based on ethanol and phosphates served as positive control, and distilled water as negative control. The whitening effects of the different formulations were tested on bovine enamel samples using a pre–post design.

## Materials and Methods

### Treatment Products and Control Group

The whitening properties of three different groups were tested as follows:

1. Test product: HAP-based (15% w/w) oral care gel (Karex gelée; Dr. Kurt Wolff GmbH & Co. KG, Bielefeld, Germany).
  Aqua, hydroxyapatite, glycerin, hydrogenated starch hydrolysate, calcium lactate, hydroxyethylcellulose PEG 40, hydrogenated castor oil, xylitol, calcium carbonate, hydroxyacetophenone, 1,2-hexanediol, caprylyl glycol, aroma, stevia rebaudiana leaf/stem powder, propylene glycol, sodium hydroxide, limonene, citral.
2. Positive control: whitening mouth rinse (Listerine Advance White, Johnson & Johnson GmbH, Neuss, Germany).
Aqua, alcohol, sorbitol, tetrapotassium pyrophosphate, pentasodium triphosphate, citric acid, poloxamer 407, sodium benzoate, eucalyptol, thymol, menthol, sodium sacharin, sodium fluoride (220 ppm F), tetrasodium pyrophosphate, propylene glycol, sucralose, aroma, disodium phosphate *.*
3. Negative control: distilled water (pH = 6.8).

### Sample preparation

Bovine incisors were cleaned and embedded in epoxy resin (EpoFix; Struers, Cleveland, Ohio, United States). The buccal enamel surface was grinded to 1,200 grit by SiC abrasive paper (Struers, Cleveland, Ohio, United States). All enamel samples were stored in distilled water for 24 hours before starting the experiments.

### Treatment with Test Product and Control Group

**Fig. 1:**
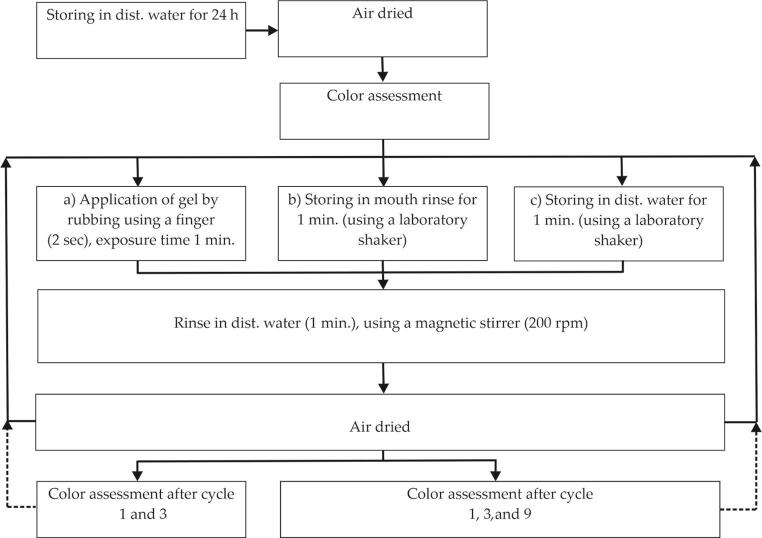
Overview of the test procedure for the analysis of ( **a** ) HAP-gel, ( **b** ) mouth rinse (positive control), and ( **c** ) distilled water (negative control). HAP, hydroxyapatite.

To ensure a comparison of the experimental data, all enamel samples were treated for 1 minute with the test products. The whole test procedure is depicted in Fig. 1 .

(a) HAP-gel: The HAP gel was applied by finger on the enamel surface (2 seconds, *n* = 8). After 1 minute, the samples were rinsed under agitation with distilled water and air dried at room temperature. The treatment procedure was repeated twice (three applications in total). Color measurements were carried out after first and third cycles.
(b) Mouth rinse and (c) control group: Enamel samples... were stored in group b (mouth rinse [ *n* = 8]) or c (distilled water [ *n* = 4]) for 1 minute by using a laboratory shaker. Afterwards the samples were rinsed under agitation with distilled water and air dried at room temperature. The treatment procedure was repeated eight times (nine applications in total). Color measurements were carried out after first, third, and ninth cycles. Nine treatment cycles were chosen for the mouth rinse testing in order to simulate a longer treatment period which is according to the manufacturers’ instructions.

### Color Measurements

Tooth color and color changes (ΔE) were analyzed by a spectrophotometer (CM-3600A, Konica Minolta Sensing Europe B.V., Bremen, Germany) using a (L*a*b*) color space with coordinates: white–black (±L*), redness–greenness (±a*), and yellow–blueness (±b*). Color changes between the different measurement and the baseline measurement in each group were calculated by using the following equation: 21 22



ΔL*= L* _after treatment_ –L* _initial_

Δa*= a* _after treatment_ –a* _initial_

Δb*= b* _after treatment_ –b* _initial_

### Statistical Analysis

Statistical analyses were conducted by one-way analysis of variance (ANOVA) with post hoc Bonferroni’s test and Levene’s test for analyses of homogeneity of variance (Origin 2019b; OriginLab Corporation Company, Northampton, Massachusetts, United States). The level of significance of α was set at ≤0.05.

### Scanning Electron Microscopy

Surface analyses of the enamel samples at baseline and after one treatment cycle were performed by scanning electron microscopy (SEM; Quanta 3D FEG scanning electron microscope, FEI Company, Hillsboro, Oregon, United States). The samples were coated with an ultra-thin carbon film by evaporation before the SEM analyses.

## Results

### Color Measurement

**Fig. 2:**
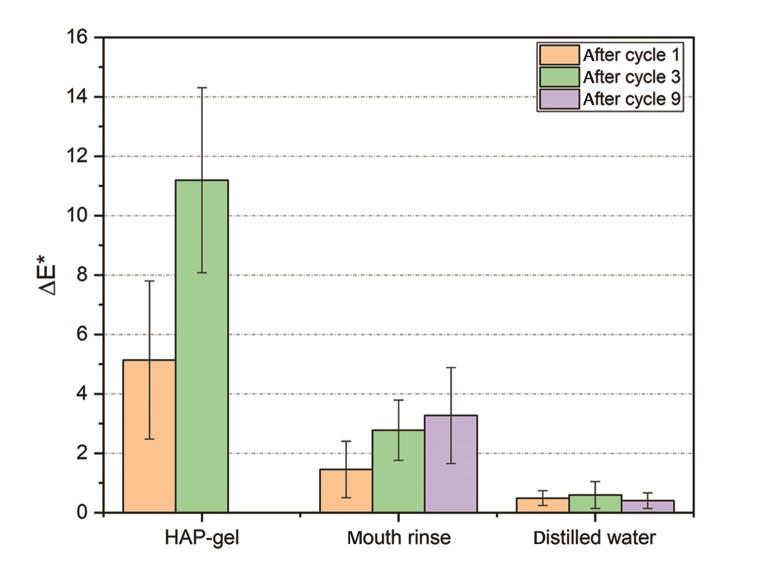
ΔE* values of the HAP-gel (group a), mouth rinse (group b), and water (group c). In ( **a** ) color measurements were performed after 1 cycle and 3 cycles, while in ( **b** ) and ( **c** ) color measurements were also performed after 9 cycles. HAP, hydroxyapatite.

Color measurement shows a significant increase in ∆E* after one cycle (5.14 [±2.66], *p* ≤ 0.006) and after three cycles (11.2 [±3.11], *p* < 0.0001) in group (a) (HAP-group) compared to group (b) (whitening mouth rinse), and group (c) (water), respectively ( Fig. 2 ). No significant increase in ∆E was measured in group (b) after one cycle. Group (b) showed an increase in ∆E after three cycles (2.77 [±1.01], *p* = 0.02) and nine cycles (ΔE = 3.27 [±1.61], *p* = 0.006) compared to group (c). A similar trend as for ∆E* could be revealed for ΔL* and Δa* ( Table 1 ). These values increased after treatment with HAP-gel (group a) and whitening mouth rinse (group b) compared to water (group c), representing an increase in brightness and reddish (minor color shift). (minor color shift). For groups (a) and (b), a correlation on ΔL* and Δa* was observed with an increasing cycle number.

**Table 1:** Mean ∆L*, ∆a*, and ∆b* values after cycle 1, 3, or 9

∆L*	After cycle 1	After cycle 3	After cycle 9
HAP-gel	4.51 [±2.48]	10.66 [±2.97]	Not performed
Mouth rinse	0.93 [±0.81]	2.31 [±1.02]	3.08 [±1.43]
Water	0.15 [±0.33]	0.32 [±0.43]	0.07 [±0.22]
∆a*			
HAP-gel	0.59 [±0.17]	1.10 [±0.19]	Not performed
Mouth rinse	0.08 [±0.13]	0.15 [±0.16]	0.29 [±0.35]
Water	0.15 [±0.33]	0.06 [±0.06]	0.01 [±0.03]
∆b*			
HAP-gel	-1.77 [±1.60]	-2.87 [±1.39]	Not performed
Mouth rinse	-0.62 [±1.00]	-0.88 [±1.19]	-0.07 [±1.12]
Water	0.16 [±0.35]	-0.29 [±0.35]	-0.23 [±0.33]
Abbreviation: HAP, hydroxyapatite.			

∆b* decreased significantly in group a, representing a decrease in yellowness of the enamel surface in the HAP-group. For groups (b) and (c), this effect could not be detected.

### Scanning Electron Microscopy

**Fig. 3:**
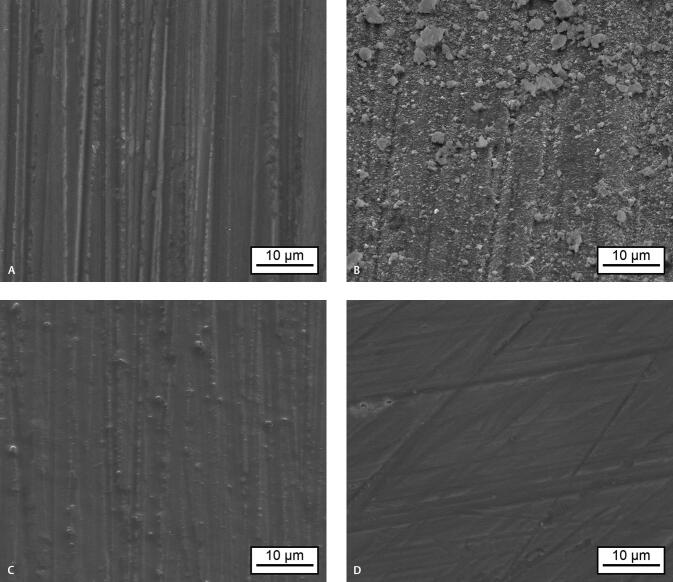
SEM images of enamel surfaces before and after treatment: ( **A** ) baseline, ( **B** ) after HAP-gel treatment, ( **C** ) after mouth rinse treatment, ( **D** ) after water treatment. HAP, hydroxyapatite; SEM, scanning electron microscope.

**Fig. 4:**
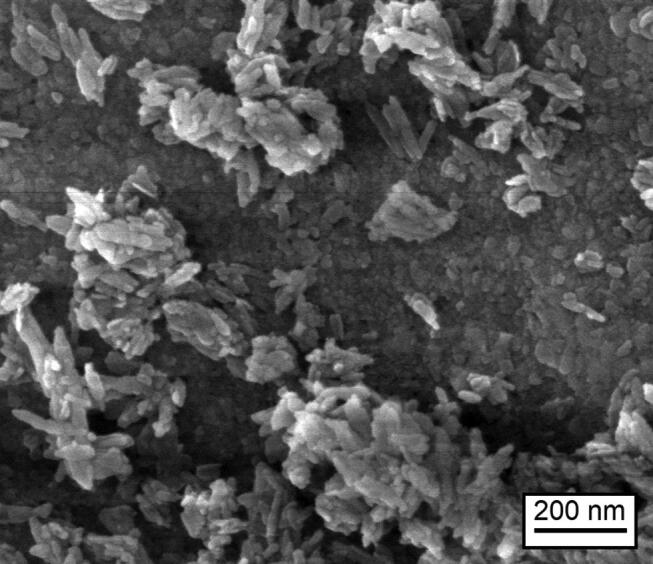
High-resolution SEM image of the deposited particles after HAP-gel treatment. HAP, hydroxyapatite; SEM, scanning electron microscope.

SEM images show that the initial enamel surface is characterized by grinding marks resulting from the sample preparation ( Fig. 3A ). After the treatment with the group (a), deposited particles were visible on the enamel surfaces ( Fig. 3B ). It can be assumed that the particles consist of HAP. High-resolution SEM images of the deposited particles revealed rod and flat shaped crystallites ( Fig. 4 ), that could be assigned to HAP since its microstructure is known and described. 10

After treatment with group (b), the enamel surface seems to be slightly smoothed by a thin layer formation compared to the initial sample surface ( Fig. 3C ). Furthermore, single particles were visible on the surface. The origin of these particles is not known. No changes could be detected after treatment with group (c) ( Fig. 3D ).

## Discussions

This in vitro study shows that the tested HAP oral gel led to an increase in ΔE, thus to a whiter tooth color already after one-time application. This effect could be enhanced by regu- larly performed applications of the gel ( Fig. 2 ). The tooth whitening properties of the HAP-gel can be explained by HAP’s adhesion to the tooth surface. Fabritius-Vilpoux et al showed in an in vitro study that HAP particles of a mouth rinse adhere to enamel surfaces; 10 this is confirmed by the SEM investigation performed in the present study. The quantity of adhesion can be increased by higher concentrations of HAP, i.e. 10% HAP showed a higher enamel coverage compared to 1 and 5% HAP. 10 Additionally, Niwa et al found a whitening optimum by using 15% HAP in a toothpaste formulation. 9 Based on these findings, the concentration of HAP was chosen to be of 15% in the tested oral care gel. Kensche et al and Lelli et al confirmed the adhesion of HAP to tooth surfaces also under in situ conditions and with an ex–in vivo study design, respectively. 23 24 Our results are in good agreement with other studies that analyzed the teeth whitening effects of HAP. 5 6 7 8 9 25 Niwa et al, for example, analyzed the in vivo whitening effect of toothpastes with 0, 3, and 15% HAP. They found that the whitening effect could be increased by higher HAP concentrations. Interestingly, in an additional in vitro experiment it was shown that the polishing properties were not altered when higher HAP concentrations were used. 9 This clearly underlines that HAP, in contrast to abrasives with a high-relative hardness (e.g. perlite, a mineral of silicate; alumina, Al _2_ O _3_ ), 20 is a suitable whitening agent which does not lead to a damage of tooth or gingiva. Moreover, HAP reduces the roughness of the teeth. 25 Dabanoglu et al and Jin et al showed that different calcium phosphates including HAP contribute to tooth whitening in vitro. 5 7 Besides the adhesion of HAP particles to the tooth surface as described above, remineralization effect of HAP may also contribute to tooth whitening (i.e. due to a smoother surface on which stains cannot attach). 9 11 26 27 An advantage of the use of the HAP in a gel formulation is the good adhesion to the tooth surface. It can be easily applied by using the finger after tooth brushing and also showed both remineralization effects 26 and erosion protective properties. 17 28 Bommer et al reported that a self-assembling peptide matrix can act as an adhesive for HAP particles improving the whitening effect of HAP alone. 6 A similar effect could be observed in HAP group of our study. The matrix of the HAP-gel may further increase the HAP-adhesion to the tooth surface compared to HAP-particles alone (which already show a good adhesion to tooth surfaces 5 10 23 ). Future studies should be carried out to analyze the whitening effect of the HAP-gel also under *in vivo* conditions. To date, only a few *in vivo* studies on the whitening effects of HAP been published. 6 9 29 *In situ* studies show an efficient reduction of bacterial colonization to enamel surfaces by using HAP-based mouth rinses. 18 23 30 This might be also an important factor for tooth whitening since stains are often incorporated into dental plaque. 1

In contrast to the HAP-gel, the tested mouth rinse showed only minor whitening effects in our in vitro setting after one, three, and nine cycles. This may be explained by the different mode of action compared to HAP. The SEM images showed that HAP adheres to the tooth surface; however, only a thin layer of unknown deposits was detectable in the mouth rinse group ( Figs. 3B 3C ). Thus, the mouth rinse may support (e.g. by its ingredients ethanol and phosphates, as well as by an acidic pH) the stain removal action of toothbrush and toothpaste, 31 but does not lead to the formation a white (protective) layer on the tooth surface. Other test approaches are necessary to understand completely the whiting mechanisms of such mouth rinses. Further testing should also involve model refinement, i.e. it would be of interest to include experimental steps in the protocol in order to examine the stability of the whitening effect against chemical (mimicking acid challenges of daily food intake) and/or mechanical (mimicking tongue and mucosa movement or saliva flow or tooth brushing) stress.

Additionally, oral-care formulations based on HAP show not only whitening properties, but was also tested to be effective in preventing cavities, improvement of gingival health, and reduction of dentin hypersensitivity. 11 Biomimetic ingredients based on HAP used in oral care show a high compatibility, since the mineral phase of the teeth and bones mainly consists of calcium and phosphate. 32 33

## Conclusion

To conclude, the *in vitro* model tested in this study can be used as basic-approach for further testing of nonabrasive whitening agents.

In this study, the nonabrasive HAP-gel showed the highest values on tooth-whitening when compared to a mouth rinse with whitening-agents. Therefore, HAP particles may be suited to be a gentle, fast, and biomimetic approach for cosmetic tooth whitening.
